# Realizing Ultrafast and Robust Sodium-Ion Storage of Iron Sulfide Enabled by Heteroatomic Doping and Regulable Interface Engineering

**DOI:** 10.3390/molecules28093757

**Published:** 2023-04-27

**Authors:** Jinke Shen, Naiteng Wu, Wei Xie, Qing Li, Donglei Guo, Jin Li, Guilong Liu, Xianming Liu, Hongyu Mi

**Affiliations:** 1 State Key Laboratory of Chemistry and Utilization of Carbon Based Energy Resources, School of Chemical Engineering and Technology, Xinjiang University, Urumqi 830017, China; sjk4011@163.com; 2 Key Laboratory of Green Energy Materials of Luoyang, College of Chemistry and Chemical Engineering, Luoyang Normal University, Luoyang 471934, Chinalijin1866@126.com (J.L.); myclxm@163.com (X.L.)

**Keywords:** N-doped Fe_7_S_8_, N, S co-doped carbon framework, ultrafast transport, ether-based electrolyte, sodium-ion batteries

## Abstract

Fe-based sulfides are a promising type of anode material for sodium-ion batteries (SIBs) due to their high theoretical capacities and affordability. However, these materials often suffer from issues such as capacity deterioration and poor conductivity during practical application. To address these challenges, an N-doped Fe_7_S_8_ anode with an N, S co-doped porous carbon framework (PPF-800) was synthesized using a template-assisted method. When serving as an anode for SIBs, it delivers a robust and ultrafast sodium storage performance, with a discharge capacity of 489 mAh g^−1^ after 500 cycles at 5 A g^−1^ and 371 mAh g^−1^ after 1000 cycles at 30 A g^−1^ in the ether-based electrolyte. This impressive performance is attributed to the combined influence of heteroatomic doping and adjustable interface engineering. The N, S co-doped carbon framework embedded with Fe_7_S_8_ nanoparticles effectively addresses the issues of volumetric expansion, reduces the impact of sodium polysulfides, improves intrinsic conductivity, and stimulates the dominant pseudocapacitive contribution (90.3% at 2 mV s^−1^). Moreover, the formation of a stable solid electrolyte interface (SEI) film by the effect of uniform pore structure in ether-based electrolyte produces a lower transfer resistance during the charge–discharge process, thereby boosting the rate performance of the electrode material. This work expands a facile strategy to optimize the electrochemical performance of other metal sulfides.

## 1. Introduction

With its usage becoming more widespread in the industry, the shortcomings of lithium resource shortage and uneven distribution have become particularly urgent. Sodium-ion batteries (SIBs) are a potential substitute for lithium on account of their similar chemical properties and ample reserves [1,2,3]. Carbon-based materials (hard carbon, graphene, etc.) [4,5], metal oxides (Fe_2_O_3_, SnO_2_, etc.) [6,7], alloys (Bi-Sb, Sb-Co, etc.) [8,9], and transition metal sulfides (FeS, MnS, etc.) [10,11] have been proposed as anode materials for the ideal SIBs with an exceptional specific capacity, excellent rate capability, and extended cycle lifespan. The transition metal sulfide anodes have attracted widespread attention owing to its weaker metal–sulfur bond, which is more propitious to the inversion reaction of sodium storage. For instance, Fe_7_S_8_ is a prospective electrode material owing to its high safety, eco friendliness, and low poisonousness as well as high theoretical capacity (610 mAh g^−1^) [12,13]. However, practical applications of Fe_7_S_8_ are still hindered by its unsatisfactory conductivity, the formation of soluble sodium polysulfides, and volume expansion. Recently, designing nanostructures, constructing heteroatom-doped composites, and encapsulating carbon are effective strategies to solve these disadvantages [14]. N doping and S doping are common methods to induce the lattice defects in a carbon structure, which provide more active sites to store the alkali metal ions and improve the intrinsic conductivity of the carbon matrix [15,16]. There are four configurations of N in materials: pyrrolic-N (N-5), pyridinic-N (N-6), graphitic-N (N-Q), and oxidized nitrogen (N-X). The five-membered ring structure of pyrrolic-N is stable and contains unbound nitrogen atoms, which can form coordination bonds and catalytic activity, and enhance the electrochemical response of electrode materials. The pyridinic-N increases the electron density of the electrode material, improving its conductivity. Graphitic-N has a small volume change and can maintain the structural stability of the material [17,18]. Except for N-X, the other configurations can improve the electrochemical performance of materials to some extent, and S doping can improve the conductivity and cycle life of the battery. For example, the nanostructure and N-doped carbon frameworks alleviate the volume expansion of spindle-like Fe_7_S_8_/N-C composites [19]. SnS/Fe_7_S_8_ bimetallic sulfide heterostructure boosts the ion/charge transfer kinetic and delivers a high first discharge/charge capacity of 873/559.2 mAh g^−1^ [20]. Fe_7_S_8_/C nanostructures with uniform hierarchical morphology exhibit outstanding sodium ion storage capabilities, retaining a capacity of 497 mAh g^−1^ even after 100 cycles at 0.1 A g^−1^, owing to the addition of a thin carbon coating [21]. These reported works indicate that it is essential to construct a rational structure with heteroatomic doping to enhance the electrochemical performance of Fe_7_S_8_/C electrode materials. In various structures, a porous structure has been considered as an optimized construction, owing to the fact that it not only increases the contact area between the active substances and the electrolyte but also alleviates the aggregation and pulverization of materials during monotonous cycles. The porous structure can usually be constructed by self-sacrificial templates. Typically, NaCl is a green and low-cost template for porous materials with a disordered and uncontrollable pore structure [22]. However, the uncontrollable pore size leads to a low tap density and the formation of extra-solid electrolyte interface (SEI) films. Furthermore, the uniform SiO_2_ and polymethyl methacrylate (PMMA) templates are generally restricted by the complicated removal process [23]. Currently, polystyrene microspheres (PS) have become one of the most popular templates due to their controllable particle size and easy removal, but their development is limited by complicated operation, a long reaction period, and the use of organic solvents in the preparation process. Therefore, it is worth seeking a green, facile, and easy-to-remove template route to construct porous Fe-based sulfide electrode materials.

**Scheme 1:**
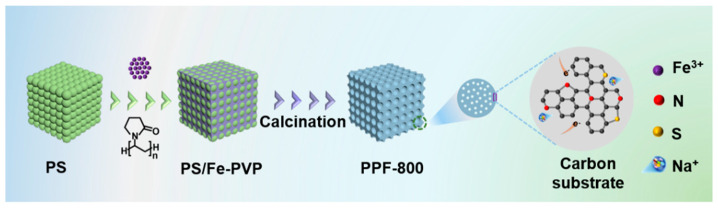
Schematic diagram of the PPF-800 preparation process.

In this work, an N-doped Fe_7_S_8_ with an N, S co-doped carbon porous framework (denoted as PPF-800) is successfully prepared by a template-assisted method. The PS template is used to form the precursor with Fe-polyvinylpyrrolidone (PVP) gel. Moreover, deionized water is employed to supersede common organic solvents as a dispersion medium in the process of template preparation, which reduces the environmental pollution and increases the safety factor. After the calcination, the N-doped Fe_7_S_8_ with a uniform pore structure is constructed (Scheme 1). The PS template with abundant hydroxyl takes the shape of the uniform pore structure and adsorbs the Fe^3+^ to form well-dispersed nanoparticles. As expected, the prepared PPF-800 electrode delivers a robust and ultrafast sodium storage performance, with a capacity of 489 mAh g^−1^ (500th)/371 mAh g^−1^ (1000th) at 5/30 A g^−1^. The outstanding electrochemical performance is attributed to the synergistic effect between the heteroatomic doping and regulable interface engineering. Heteroatomic doping in Fe_7_S_8_ nanoparticles and carbon frameworks is propitious to accelerate the Na^+^/electron conveyed through the enhanced intrinsic conductivity and capacitive contribution (90.3% at 2 mV s^−1^). Moreover, the well-dispersed Fe_7_S_8_ nanoparticles in the carbon framework endow the electrode with the capability to alleviate volumetric expansion and dissolution of sodium polysulfides. More importantly, combined with the uniform pore structure and ether-based electrolyte, a regulable interface with small transfer resistance is formed during the charge–discharge process to further improve the rate performance of the electrode material.

## 2. Results

**Figure 1:**
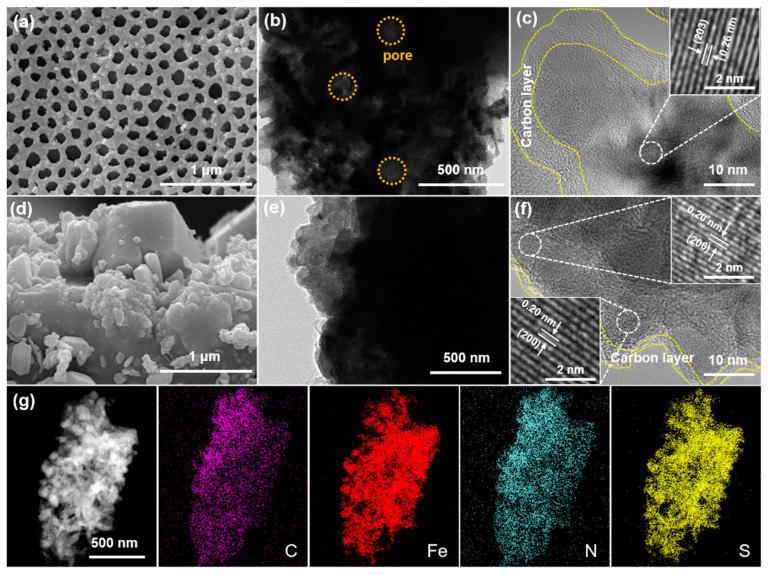
SEM and TEM images of (**a**–**c**) PPF-800 and (**d**–**f**) PF-800. (**g**) EDS elemental mapping images of PPF-800.

The morphologies of the as-synthesized samples were characterized by SEM and TEM. PPF-800 exhibits uniformly distributed pores with a diameter of 140–200 nm (Figure 1a). The porous structure can also be examined by TEM image (Figure 1b) and cross-section SEM images of the pole piece (Figure S1, Supplementary Materials), which is consistent with the SEM images. The (203) plane of hexagonal Fe_7_S_8_ is clearly depicted in the high-resolution TEM (HRTEM) image shown in Figure 1c, revealing parallel and distinct lattice fringes with a lattice spacing of 0.26 nm. In addition, EDX elemental mapping (Figure 1g) reveals the homogenous distribution of C, Fe, N, and S elements, which implied the uniform doping of nitrogen in the PPF-800. As the counterpart, the sample synthesized without a PS template (denoted as PF-800) exhibits bulk and irregular particles without visible holes (Figure 1d). The HRTEM image (Figure 1e) displays lattice fringes with a *d*-spacing of 0.29 nm and 0.20 nm, agreeing with the (200) and (206) planes of Fe_7_S_8_ (Figure 1f).

**Figure 2:**
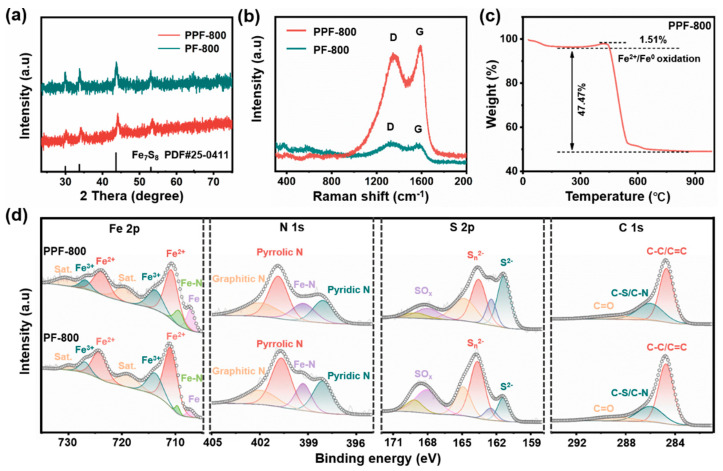
(**a**) XRD patterns and (**b**) Raman spectra of PPF−800 and PF-800. (**c**) TG curve of PPF-800. (**d**) XPS spectra of Fe 2p, N 1s, S 2p, and C 1s.

The composition and crystalline structure of the samples were determined by XRD (Figure 2a). Both PPF-800 and PF-800 show similar and dominant diffraction peaks at about 29.95°, 33.75°, 43.65°, and 53.07°, which can be assigned to the (200), (203), (206), and (220) planes of the hexagonal phase Fe_7_S_8_ crystal (JCPDS No. 25-0411), respectively. All the prepared samples show right-shifted diffraction peaks compared with the XRD standard pattern, indicating that the lattice parameters are reduced [24]. Based on previous reports, this may be caused by N doping, which can induce electronic modulation, and S^2−^ may be replaced by N^3−^ ions to form Fe-N during the reaction [25]. The Raman spectra of the samples (Figure 2b) display two peaks of carbon at 1360 cm^−1^ and 1590 cm^−1^, which are ascribed to the disordered carbon (D-band) and graphitic carbon (G-band) [26,27], respectively. The peak intensity ratio (*I*_D_/*I*_G_) of PPF-800 (0.95) is lower than that of PF-800 (1.04), suggesting a higher degree of graphitization in PPF-800, which is beneficial for improving electrical conductivity [28,29]. Moreover, the percentage composition of the carbon matrix in the samples was determined through TG analysis under air atmosphere. Figure 2c and Figure S3 show that the samples have slight mass loss between 30 °C and 150 °C due to the evaporation of water. A minor mass increases from 250 to 400 °C due to the oxidation of Fe^0^/Fe^2+^ to Fe^3+^. The weight loss after 450 °C was mainly due to the combustion of the carbon matrix along with the oxidation of Fe_7_S_8_. According to the TG results, the content of the carbon matrix in PPF-800 can be estimated as 33.9%, which is similar to that of the counterpart (29.3%). Furthermore, X-ray photoelectron spectroscopy (XPS) was utilized to investigate the chemical makeup and valence state of surface elements in the samples. Figure S2 shows the full XPS spectrum exhibiting that Fe, S, C, and N coexist in the samples. The Fe 2p spectra (Figure 2d) demonstrate that all samples exhibit a pair of broad satellite peaks and 2 pairs of well-defined peaks located at 710.8/724.1 and 713.8/726.9 eV, respectively, which correspond to the Fe^2+^ 2p_3/2_/2p_1/2_ and Fe^3+^ 2p_3/2_/2p_1/2_ states [30,31,32,33]. There are also two peaks detected at 706.3 eV and 709.4 eV, which are likely associated with Fe^0^ and Fe-N. The peak observed in Fe^0^ may be caused by a carbothermal reduction reaction during the high-temperature vulcanization process [34]. The existence of Fe-N indicates that nitrogen has been successfully doped in the Fe_7_S_8_ anode. The formed Fe-N bonds would not only modulate the electronic structure of Fe_7_S_8_ to accelerate ion transport but also catalyze the reaction between Fe_7_S_8_ and Na^+^ to boost the charge storage kinetics. It is worth noting that XPS results show that the contents of Fe-N and Fe^0^ in PPF-800 are higher than those of PF-800, which is attributed to the presence of the PS template. The uniformly distributed Fe^3+^ on the surface of the PS template prefers to promote the reduction in iron ions and generate more Fe-N bonds. The N 1s spectra reveal the existence of pyridinic-N (398.1 eV), pyrrolic-N (400.8 eV), graphitic-N (401.34 eV), and Fe-N (399.3 eV) [35,36,37,38]. In the spectra of S 2p, there are 3 pairs of distinct peaks located at 161.4/162.4 eV, 163.6/164.8 eV, and 168/169.2 eV, corresponding to the S^2−^ 2p_3/2_/2p_1/2_, S_n_^2−^ 2p_3/2_/2p_1/2_, and SO_x_ 2p_3/2_/2p_1/2_, respectively [34,35,39]. The C1s spectrum exhibits three peaks at 284.8 eV, 286 eV, and 289 eV, which are assigned to the C-C/C=C, C-S/C-N and C=O [25], confirming the formation of the nitrogen and sulfur co-doped carbon matrix. The N, S co-doped carbon framework endows PPF-800 with a good physical adsorption effect on sodium polysulfide, which alleviates the shuttle effect of polysulfide to improve the electrochemical performances [40,41].

**Figure 3:**
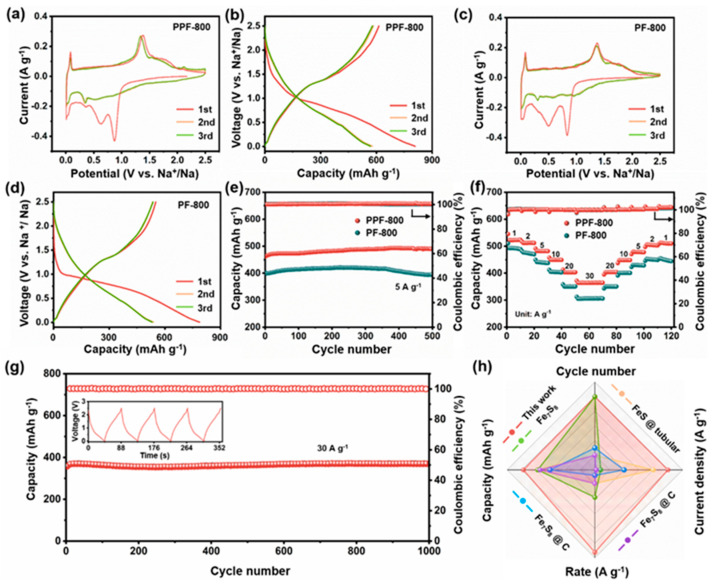
CV curves of (**a**) PPF-800 and (**c**) PF-800 at the scanning rate of 0.1 mV s^−1^. Initial three discharge–charge curves of (**b**) PPF-800 and (**d**) PF-800. (**e**) Cycling performance under the current density of 5 A g^−1^; (**f**) rate performance of PPF-800 and PF-800. (**g**) Long-term cycling performance of PPF-800 at 30 A g^−1^. (**h**) Comparison of electrochemical properties between PPF-800 and reported Fe-based anodes.

Constant current discharge/charge and cyclic voltammetry (CV) tests were conducted in the voltage range of 0.005–2.5 V (vs. Na^+^/Na) to evaluate the electrochemical performance. Figure 3a displays the CV profiles for the first 3 cycles at 0.1 mV s^−1^. During the first cathodic process, two peaks were observed at 0.90 V and 0.62 V, respectively. These peaks represent the intercalation of Na^+^ into Fe_7_S_8_ (Equation (1)) and the formation of the solid electrolyte interface (SEI) film. These peaks gradually disappear, which is ascribed to the irreversibility of the SEI formation process. The reduction peak at 0.33 V manifests that Fe^2+^/Fe^3+^ are converted into Fe^0^ and formed Fe and Na_2_S (Equation (2)) [42]. During the subsequent anodic process, the oxidation peaks at 1.38 V, 1.54 V, and 2.11 V are connected to the desodiation processes that lead to the Na_2_FeS_2_ (Equation (3)), Na_2−x_FeS_2_ (Equation (4)), and Fe_7_S_8_ (Equation (5)) [43], respectively. The peaks at 0.05 (cathodic scan) and 0.076 V (anodic scan) are caused by the solvated-Na^+^ co-intercalation/deintercalation in the carbon matrix in the ether-based electrolyte. Moreover, CV curves of PF-800 (Figure 3c) are consistent with the typical sodiation/desodiation behavior of PPF-800. The almost overlapping CV curves show that the materials have reliable reversibility and stability after the first sodiation.

Discharge: Fe_7_S_8_ + 8Na^+^ + 8e^−^ → 4Na_2_FeS_2_ + 3Fe (1)

Na_2_FeS_2_ + 2Na^+^ + 2e^−^ → 2Na_2_S + Fe (2)

Charge: 2Na_2_S + Fe → Na_2_FeS_2_ + 2Na^+^ + 2e^−^ (3)

Na_2_FeS_2_ → Na_2−x_FeS_2_ + *x*Na^+^ + *x*e^−^ (4)

4Na_2_FeS_2_ + 3Fe → Fe_7_S_8_ + 8e^−^ + 8Na (5)

As depicted in Figure 3b, the PPF-800 exhibits a high-discharge capacity (808 mAh g^−1^) and a high-charge capacity (613 mAh g^−1^) in the first cycle at 0.05 A g^−1^, with the initial Coulombic efficiency (ICE) of 75.86%, which is higher than that of PF-800 (70.02%) (Figure 3d). The irreversible capacity may be attributable to the partial dissolution of polysulfide and the subsequent formation of SEI films through the decomposition of the surface electrolyte [44]. Figure 3f depicts the rate performance of two anode materials at different current densities. PPF-800 delivers the higher discharge capacities of 523 mAh g^−1^, 511 mAh g^−1^, 481 mAh g^−1^, 449 mAh g^−1^, 403 mAh g^−1^, and 364 mAh g^−1^ at 1 A g^−1^, 2 A g^−1^, 5 A g^−1^, 10 A g^−1^, 20 A g^−1^, and 30 A g^−1^, respectively. Notably, the cell can still maintain the stable reversible capacity, when gradually changed back to smaller current densities. In the evaluation of cycling stability (Figure 3e), PPF-800 displays a superior sodium storage capacity of 489 mAh g^−1^ after 500 cycles at 5 A g^−1^ (capacity retention ≈102%). For a comparison, the PF-800 electrode without the PS template shows slight capacity fading (394 mAh g^−1^ after 500 cycles). However, both PPF-800 and PF-800 electrodes show the more stable cyclic performance at high-current density than that of the porous carbon (Figure S4), which indicates that the uniform dispersed Fe_7_S_8_ nanoparticles in the carbon substrate provide the dominating Na^+^ storage capability. The porous structure with the N, S co-doped carbon matrix ensures a high degree of utilization of active materials and promotes the rapid electron/ion migration of nanoparticles. Furthermore, the reversible capacity still reserves 371 mAh g^−1^ after 1000 cycles at 30 A g^−1^ of PPF-800 (Figure 3g), manifesting the ultrafast and excellent stability sodium storage performance. Impressively, only 44.4 s was spent on each charge process, which promises to relieve anxiety about slow charging. Moreover, the electrochemical performance of the PPF-800 electrode is also superior to the reported iron sulfide electrodes both in specific capacity and cyclic stability (Figure 3h and Table S1) [38,45,46,47].

**Figure 4:**
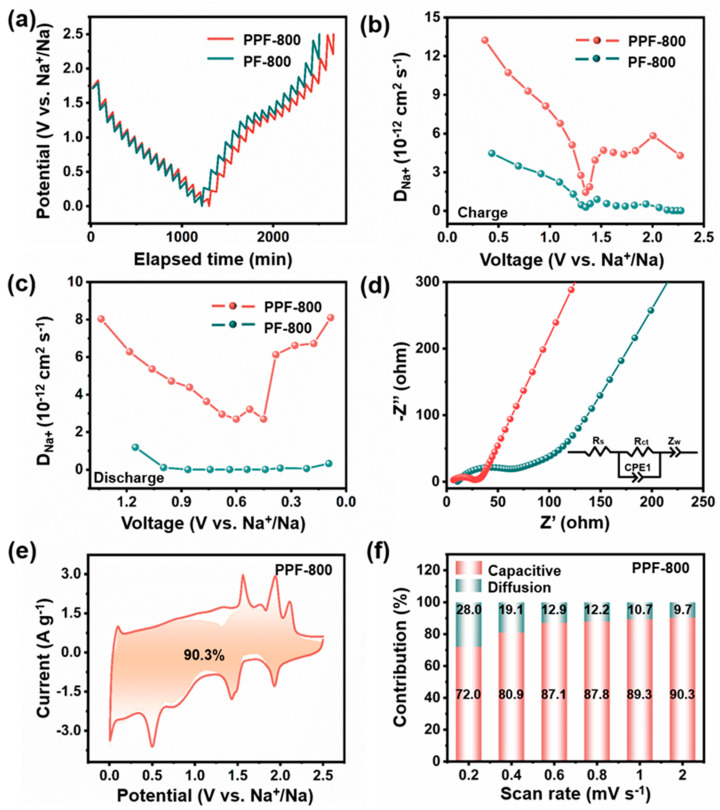
(**a**) GITT curves, (**b**) *D*_Na+_ at charge states, (**c**) *D*_Na+_ at discharge states, and (**d**) Nyquist plots of PPF-800 and PF-800. (**e**) The capacitive contribution to the charge storage at 2 mV s^−1^; (**f**) the percentage of capacitive contributions at different scan rates of PPF-800.

To comprehend the superior rate performance of the fabricated samples, GITT, EIS, and CV measurements were carried out. GITT methods were studied to compute the Na^+^ diffusion coefficient of the as-prepared samples during the charging and discharging processes. Under a pulse current of 100 mA g^−1^, the corresponding voltage–time curves were obtained during the 20 min galvanostatic discharge/charge tests with a rest interval of 60 min (Figure 4a). The diffusion coefficient of sodium ion (*D*_Na+_) for the two electrodes can be calculated according to Equation (6):

(6) $$D_{GITT}=\frac{4}{\pi \tau }{\left(\frac{m_{B}V_{M}}{M_{B}S}\right)}^{2}{\left(\frac{\Delta E_{s}}{\Delta E_{t}}\right)}^{2}$$

In this equation, *τ*, *m*, *M*, *V*_m_, and *S* represent the current pulse time, mass, molar mass, molar volume, and electrode surface area of the electrode material, respectively [48,49]. ∆*E*_τ_ is the change in battery voltage during the pulse, and ∆*E*_s_ is the voltage difference between the initial and steady state [50]. In general, all electrodes show the same variation tendency, and the range of calculated *D*_Na+_ values is 10^−13^–10^−12^ cm^2^ s^−1^, and the values of PPF-800 are higher than PF-800 in the whole capacity measured (Figure 4b,c), which is agreement with its rate performance and cycling stability over extended periods of time. Meanwhile, this outcome is supported by the results of EIS tests. As demonstrated in Figure 4d, the Nyquist plots of two electrodes exhibit a high-frequency semicircle region and low-frequency lines, which are associated with the resistance of electrolyte solution (*R*_s_), the charge-transfer resistance (*R*_ct_), and the Warburg impedance (*W*) [51]. Based on the impedance parameters obtained from fitting (Table S2), the lower *R*_ct_ of PPF-800 indicates that the porous structure reduces the resistance of the electrode/electrolyte interface, thereby facilitating the charge transfer processes. The reaction kinetics and charge storage mechanism were further studied by CV measurements at different scan rates (0.2 mV s^−1^, 0.4 mV s^−1^, 0.6 mV s^−1^, 0.8 mV s^−1^, 1 mV s^−1^, and 2 mV s^−1^, Figure S5a,b). Figure 4f and Figure S5c show that the proportion of capacitive contributions gradually increases in PPF-800 and PF-800. Impressively, the capacitive contribution of PPF-800 (90.3%) electrode at 2 mV s^−1^ (Figure 4e, yellow-shaded region) is much higher than that of PF-800 (79.76%, Figure S5d). The improved kinetic behavior of PPF-800 demonstrates that the heteroatomic doping and regular porous structure would induce more pseudocapacitive contribution, further obtaining the robust and fast sodium storage performance.

**Figure 5:**
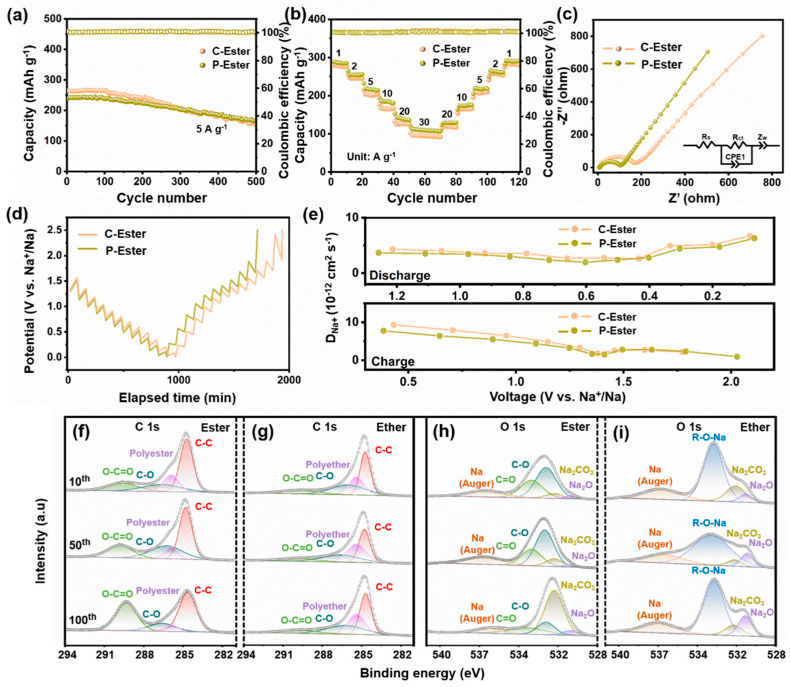
(**a**) Cycling performance under the current density of 5 A g^−1^, (**b**) rate performances, (**c**) Nyquist plots, (**d**) GITT curves, and (**e**) *D*_Na+_ at charge/discharge states of C-Ester and P-Ester. (**f**–**i**) Ester/ether-based electrolyte XPS spectra of C 1s and O 1s.

To investigate the impact of diverse interfaces between the electrolyte and electrode, PPF-800 was further tested in an ester-based electrolyte. As shown in Figure 5a, the cells cycled at 5 A g^−1^ in the ester-based electrolyte with 1 M NaClO_4_ (C-Ester) and ester-based electrolyte with 1 M NaPF_4_ (P-Ester) only deliver the reversible capacities of 158 and 166 mAh g^−1^ after 500 cycles, respectively. Moreover, the ester-based electrolyte exhibits considerably lower capacity retention (59.8%/69.7%) compared with the ether-based electrolyte (102%). The rate performance of C-Ester and P-Ester is also inferior to that of PPF-800 in the ether-based electrolyte at the same current density. Notably, the electrochemical performance of PPF-800 in ester electrolytes was not affected by the change in electrolyte salts. In addition, the CV curves not only present the similar peak shapes of C-Ester (Figure S6a) and P-Ester (Figure S6d), but also process the approximate capacitive contribution at different scanning rates (Figure S6b,e). As shown in Figure S6c,f, the capacitive contribution of C-Ester and P-Ester at 2 mV s^−1^ is 86.6% and 86.5%, respectively.

The GITT test also indicated similar *D*_Na+_ values during the charging and discharging process (Figure 5d,e). The kinetics analyses demonstrated that the electrochemical behaviors of electrodes would not be affected by the choice of sodium salts in the electrolytes, in agreement with previous findings [52]. Metal sulfide anodes usually deliver a superior electrochemical performance in ether-based electrolytes, which would be attributed to the difference in the components and thickness of the formed SEI films. To prove this conjecture, XPS analysis was performed to examine the surface states of the cycled electrodes (10th, 50th, and 100th cycle) in both ether and ester-based electrolytes. In the ether-based electrolyte, the C 1s spectrum (Figure 5f,g) was analyzed and resolved into four components. The peaks observed at 284.8 eV, 286 eV, 289.9 eV, and 285.4 eV were identified as C-C, C-O, O-C=O and polyethers, respectively [53,54]. The O 1s spectrum (Figure 5h,i) shows the R-O-Na (532.6 eV), Na_2_CO_3_ (531.3 eV), Na_2_O (530.0 eV), and Na (Auger) (536.3 eV) peaks [54,55,56]. The ester-based electrolyte exhibits a peak for polyesters at 285.9 eV, while a separate peak at 533.0 eV corresponds to C=O bonds. Sodium alkoxides (RCH_2_ONa) that originate from the reduction in diglyme and Na_2_CO_3_ in the ether-based electrolyte are responsible for the C-O and O-C=O groups. In contrast, C-O and O-C=O are consistent with sodium alkylcarbonates (ROCO_2_Na) and Na_2_CO_3_ in the ester-based electrolyte. As shown in Figure 5f, the intensity of the C-O and O-C=O peaks increases with the cycles, which indicates that the SEI film gradually grows thicker during the cycles. However, this trend is absent in the ether-based electrolyte, indicating the formation of stable SEI films (Figure 5g). It is reported that the unstable ROCO_2_Na would be partially decomposed into RCH_2_ONa and Na_2_CO_3_, which leads to the formation of an unstable SEI layer in an ester-based electrolyte [57]. The impedance fitting results (Figure 5c) agree well with XPS analyses. The R_ct_ of the ester-based electrolyte (167.4 Ω/97.55 Ω) is significantly higher than that of the ether-based electrolyte (20.77 Ω), demonstrating that an unstable SEI film was generated in the ester-based electrolyte. As a result, it is essential for the ether-based electrolyte to regulate the interface between the electrode and electrolyte in advanced SIBs.

## 3. Materials and Methods

### 3.1. Materials Preparation

All raw materials were purchased from Shanghai Macklin Biochemical Technology Co., Ltd, Shanghai, China.

#### 3.1.1. Synthesis of Polystyrene (PS)

Typically, we added 5.3 mL of styrene into 45 mL of deionized water containing 0.92 g of polyvinylpyrrolidone (PVP, average MW = 10,000) and subjected the mixture to magnetic stirring for 30 min. Then, we dissolved 0.49 g of ammonium persulfate (APS) in 10 mL of deionized water, which was added to the above solution with continuous stirring at 70 °C for 6 h. After cooling in an ice bath following the reaction, the white solution was collected and washed with deionized water. Finally, the solution was dried at 100 °C overnight.

#### 3.1.2. Synthesis of PPF-800

An amount of 0.6 g PS, 0.5 g PVP (average MW = 1,300,000), and 4 mmol Fe(NO_3_)_3_⋅9H_2_O were dissolved in 40 mL of deionized water. Then, the mixture solution was agitated at 80 °C in a water bath to form a gel and dried in air at 80 °C for 12 h. Afterwards, the dried gel mixture and thioacetamide (TAA) were loaded in the alumina boat (1:10), followed by annealing at 800 °C for 3 h under an Ar/H_2_ atmosphere.

#### 3.1.3. Synthesis of PF-800 and PC

For comparison, PF-800 and PC were prepared through the same processes, except that PS was not added to PF-800, and Fe(NO_3_)_3_⋅9H_2_O was not involved in PC.

### 3.2. Materials Characterizations

The X-ray diffraction (XRD) analysis was performed on a Bruker D8 (Bruker Company, Billerica, USA) with Cu Kα radiation to determine the crystal structure of the samples. The morphology and structures of the as-prepared samples were characterized by a scanning electron microscope (SEM, Sigma 500, Zeiss Company, Oberkochen, Germany) and a transmission electron microscope (TEM, Talos F200S, 200 kV, FEI Company, Hillsboro, USA). X-ray photoelectron spectroscopy with Al Kα radiation (XPS, K-Alpha, Thermo Fisher Scientific, Waltham, USA) was carried out to determine the valence state of the samples. The XPS calibration procedure was based on C1s 284.8 eV. The thermal gravimetric analysis was performed on a thermal analyzer (SII TG/DTA6300, Seiko Company, Tokyo, Japan) in air. The presence of carbon was investigated by using a Raman spectrometer with an excitation laser beam wavelength of 633 nm (LabRAM Aramis, HORIBA Jobin-Yvon Company, Paris, France)

### 3.3. Electrochemical Measurements

The working electrode was manufactured by dispersing active material, carbon black (conductive agent), and polyvinylidene fluoride (PVDF, binder) in *N*-methyl-2-pyrrolidone (NMP) solvent at a mass ratio of 7:2:1 to form a uniform slurry. Then, the slurry was coated on the copper foil substrate and dried at 100 °C in a vacuum for 12 h. The electrode was cut into discs with a diameter of 12 mm (active mass loading in the range of 1.1–1.2 mg cm^−2^), the glass fiber paper (Whatman GF/D, Cytiva) was applied as the separators, and each coin cell was added to 200 μL of electrolyte. Then, the electrode, electrolyte, separator, and Na foil were assembled into CR-2032 coin-type batteries in a glove box filled with argon for the electrochemical performance test. The three types of electrolytes were employed, which are 1 M NaPF6 in DIGLYME; 1 M NaClO_4_ in a mixture of ethylene carbonate (EC), dimethyl carbonate (DMC), and diethyl carbonate (DEC) (volume 1:1:1); and 1 M NaPF_4_ in a mixture of ethylene carbonate (EC), dimethyl carbonate (DMC), and diethyl carbonate (DEC) (volume 1:1:1). The galvanostatic charge/discharge were measured on a Neware apparatus (Shen Zhen, CT-3008W). The electrochemical impedance spectra (EIS) of the cells were carried out in the frequency range of 100 KHz to 0.01 Hz at an AC amplitude of 5 mV on a Parstat 4000+ electrochemical workstation. Cyclic voltammetry (CV) measurements were also performed on the Parstat 4000+ workstation in the potential range from 0.005 to 2.5 V (vs. Na^+^/Na) with different scanning rates. During the typical Galvanostatic Intermittent Titration Technique (GITT) measurement, the battery was charged and discharged with a pulse time (τ) of 20 min under a 100 mA g^−1^ pulse current density, then followed by a relaxation time of 60 min. Moreover, all the capacities were obtained based on the entire Fe_7_S_8_-PPF-800/Fe_7_S_8_-PF-800 composites.

## 4. Conclusions

In brief, the N-doped Fe_7_S_8_ nanoparticles in the N, S co-doped porous carbon framework was successfully created by the template-assisted method. This order of porous structure shortens the Na^+^/electron diffusion path and alleviates the volume expansion and shuttle effect of sodium polysulfides. Benefiting from the purposeful doping and interfacial regulation, PPF-800 delivers a high capacitive contribution (90.3% at 2 mV s^−1^) and outstanding cycle stability, with a discharge capacity of 489 mAh g^−1^ after 500 cycles at 5 A g^−1^ and a reversible capacity as high as 371 mAh g^−1^ at a high-current density (30 A g^−1^) after 1000 cycles in the ether-based electrolyte. XPS and kinetic analyses show that the ether-based electrolyte produces a more stable SEI film than the ester-based electrolyte, which can reduce the Na^+^/electron transfer resistance during the sodiation/desodiation process and further guarantee the excellent rate performance of the electrode material. This work provides a simple and economical route to prepare porous materials for promising sodium-ion battery anodes.

## Data Availability

The data presented in this study are available on request from the corresponding author.

## Supplementary Materials

The following supporting information can be downloaded at: https://www.mdpi.com/article/10.3390/molecules28093757/s1, Figure S1: Cross-section SEM images of pole piece; Figure S2: The XPS survey spectra of PPF-800 and PF-800; Figure S3: TG curve of Sn-BDC; Figure S4: (**a**) Cycling performance of PC at the current density of 5 A g^−1^. (**b**) Rate capability at various densities; Figure S5: CV curves at different scan rates of (**a**) PPF-800 and (**b**) PF-800. (**c**) Capacitive contribution to the charge storage at 2 mV s^−1^ and (**d**) percentage of capacitive contributions at different scan rates of PPF-800; Figure S6: (**a**,**d**) CV curves at different scan rates, (**b**,**e**) percentage of capacitive contributions at different scan rates, and (**c**,**f**) capacitive contribution to the charge storage at 2 mV s^−1^ of C-Ester and P-Ester; Table S1: Electrochemical performance comparison of PPF-800 and reported Fe_7_S_8_; Table S2: The fitting values of Nyquist plots after 100 cycles.

## Sample Availability

Samples of the compounds are available from the corresponding author Naiteng Wu.
