# Supplementary material for: Realizing Ultrafast and Robust Sodium-Ion Storage of Iron Sulfide Enabled by Heteroatomic Doping and Regulable Interface Engineering
Source: Molecules. 2023 Apr 27;28(9):3757. doi: 10.3390/molecules28093757 (PMC10180235; doi:10.3390/molecules28093757)
Supplement: Supplementary file 1 [file molecules-28-03757-s001.zip › molecules-2326555-supplementary.pdf]

Supplementary Materials

1. Supplemented Figures

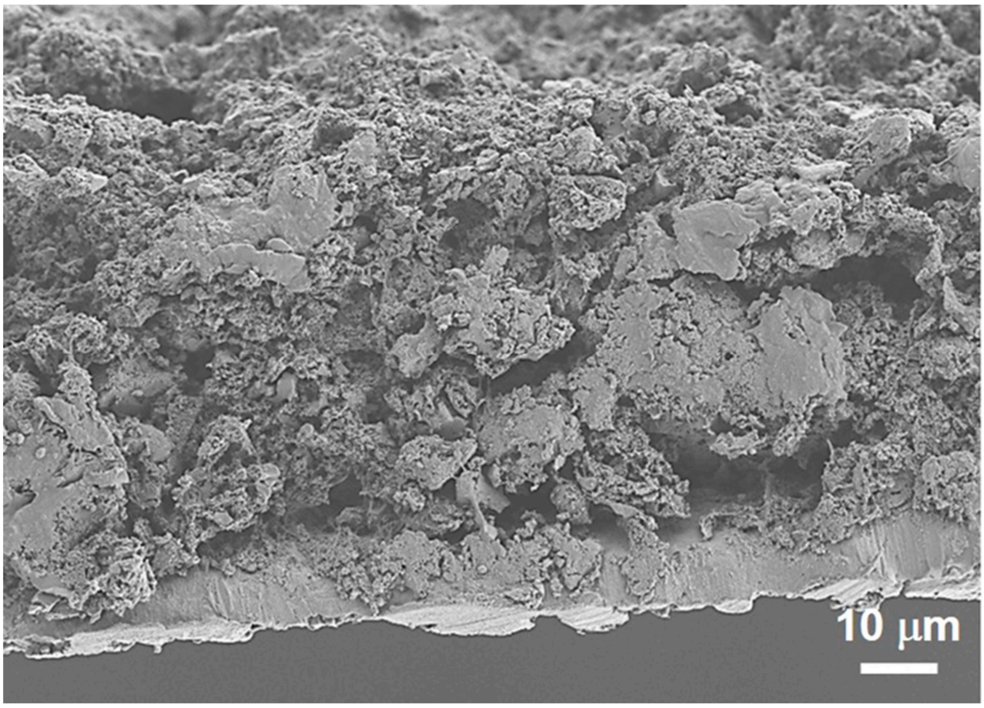

Figure S1. Cross-section SEM images of pole piece

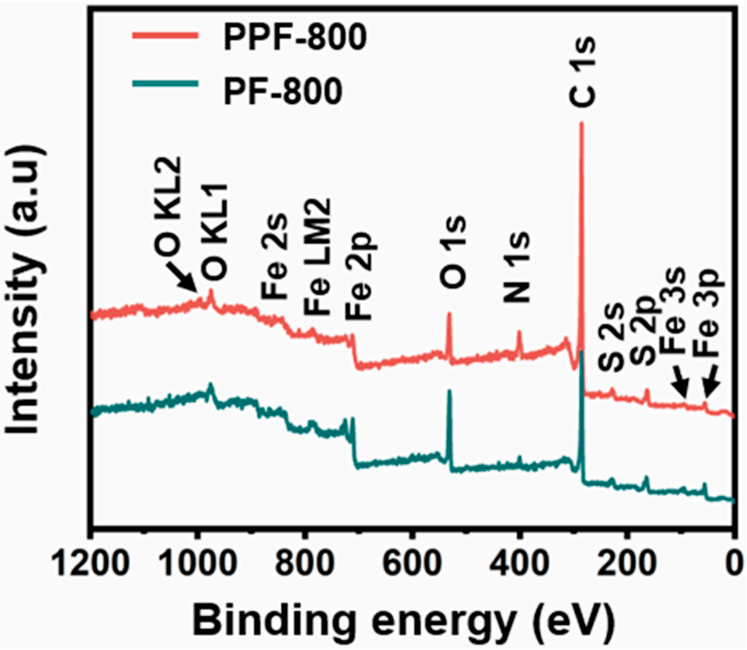

Figure S2. The XPS survey spectra of PPF-800 and PF-800

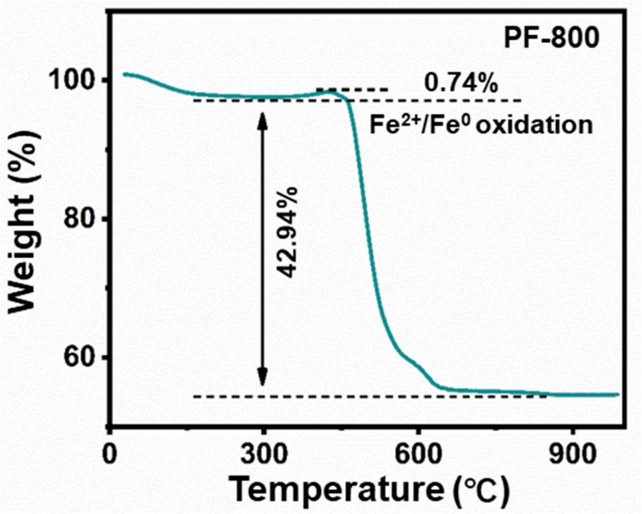

Figure S3. TG curve of Sn-BDC

The chemical reaction equation of pyrolysis process in air is as follows:

$$4Fe_7S_8 + 53O_2 = 14Fe_2O_3 + 32SO_2$$

The weight loss of the sample (W_Z) mainly comes from the evaporation of water vapor (W_{H_2O}), carbon combustion (W_C) and the change of mass in the oxidation process from Fe_7S_8 to Fe_2O_3 (W_F). The calculated equation was described as follows:

PPF-800:

$$W_C = W_Z - W_{H_2O} - W_F = W_Z - W_{H_2O} - \left(1 - \frac{14 \times M_{Fe_2O_3}}{4 \times M_{Fe_7S_8}}\right) = 33.9\%$$

PPF-800:

$$W_C = W_Z - W_{H_2O} - W_F = W_Z - W_{H_2O} - \left(1 - \frac{14 \times M_{Fe_2O_3}}{4 \times M_{Fe_7S_8}}\right) = 29.3\%$$

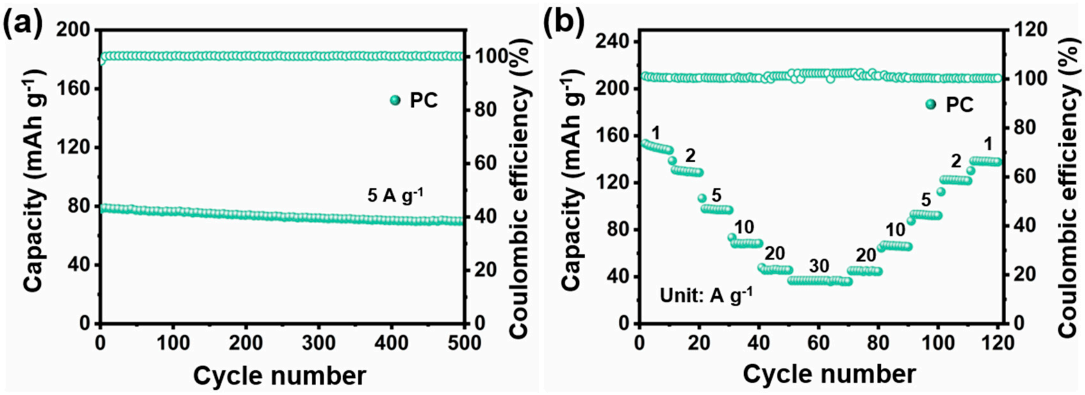

Figure S4. (a) Cycling performance of PC at the current density of 5 A g^{-1}. (b) Rate capability at various densities.

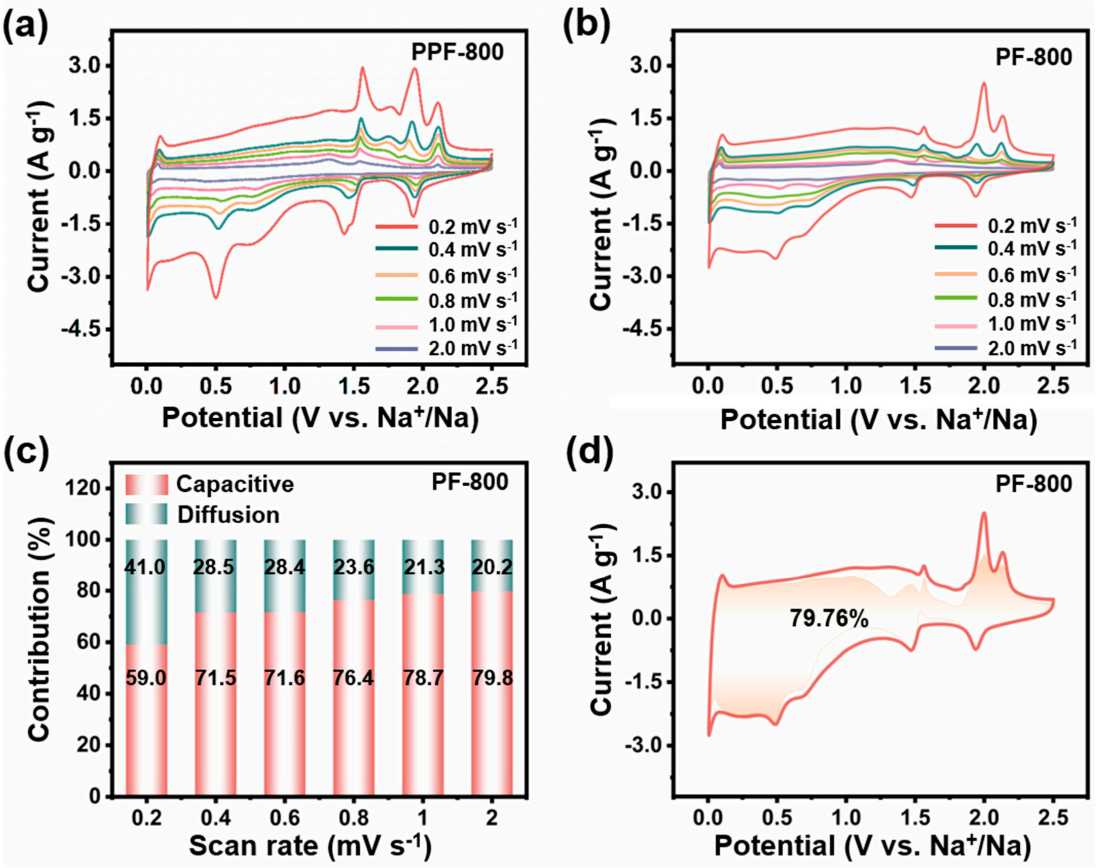

Figure S5. CV curves at different scan rates of (a) PPF-800 and (b) PF-800. (c) Capacitive contribution to the charge storage at 2 mV s^{-1}, (d) percentage of capacitive contributions at different scan rates of PPF-800.

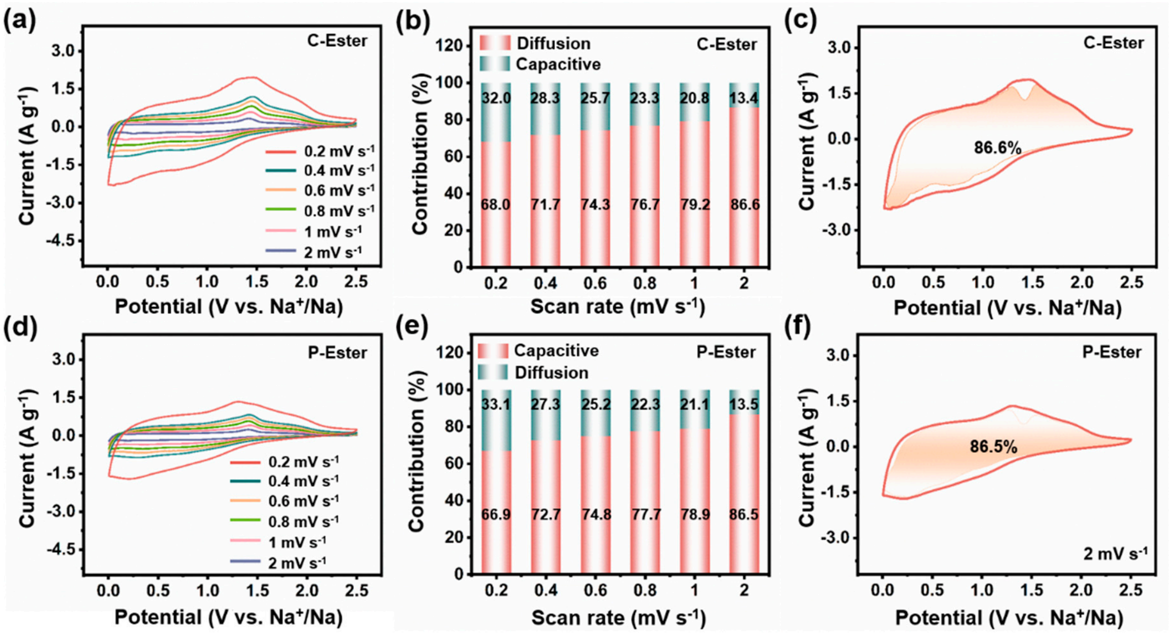

Figure S6. (**a** and **d**) CV curves at different scan rate, (**b** and **e**) percentage of capacitive contributions at different scan rates, (**c** and **f**) capacitive contribution to the charge storage at 2 mV s^{-1} of C-Ester and P-Ester.

Table S1. Electrochemical performance comparison of PPF-800 and reported Fe_7S_8

**Anode materials**	**Current density (A g^{-1})**	**Cycled capacity (mAh g-1)**	**Cycles**	**Rate (A g^{-1})**	**Ref**
**Fe_7S_8**	0.4	391.3	500	10	S1
**Fe_7S_8@C**	0.1	375	100	5	S2
**Fe_7S_8@tubular**	4	391	100	5	S3
**Fe_7S_8@C**	2	306	150	2	S4
**PPF-800**	5	489	500	30	Our work

Table S2. The fitting values of Nyquist plots after 100 cycles

	**Rs (Ω)**	**Rct (Ω)**
**PPF-800**	5.07	20.77
**PF-800**	9.98	37.15
**C-Ester**	6.574	167.4
**P-Ester**	7.697	97.55

References

S1. Jiang, F.; Wang, Q.; Du R.; Yan, X.; Zhou, Y. Fe_7S_8 nanoparticles attached carbon networks as anode materials for both lithium and sodium ion batteries. *Chem. Phys. Lett*. **2018**, *706*, 273–279.

S2. He, Q.; Rui, K.; Yang, J.; Wen, Z. Fe_7S_8 Nanoparticles Anchored on Nitrogen-Doped Graphene Nanosheets as Anode Materials for High-Performance Sodium-Ion Batteries. *ACS Appl. Mater. Inter.* **2018**, *10*, 29476–29485.

S3. Cao, Z.; Ma, X.; Dong, W.; Wang, H. FeS@tubular mesoporous carbon as high capacity and long cycle life anode materials for lithium- and sodium-ions batteries. *J. Alloy. Compd*. **2019**, *786*, 523–529.

S4. Huang, W.; Sun, H.; Shangguan, H.; Cao, X.; Xiao, X.; Shen, F.; Mølhave, K.; Ci, L.; Si, P.; Zhang, J. Three-dimensional iron sulfide-carbon interlocked graphene composites for high-performance sodium-ion storage. *Nanoscale* **2018**, *10*, 7851–7859.
